# Functional Characterization of the Oxantel-Sensitive Acetylcholine Receptor from *Trichuris muris*

**DOI:** 10.3390/ph14070698

**Published:** 2021-07-20

**Authors:** Tina V. A. Hansen, Richard K. Grencis, Mohamed Issouf, Cédric Neveu, Claude L. Charvet

**Affiliations:** 1 INRAE, Université de Tours, ISP, F-37380 Nouzilly, France; tvalstrup@icloud.com (T.V.A.H.); sarl.maybiotech@gmail.com (M.I.); cedric.neveu@inrae.fr (C.N.); 2 Lydia Becker Institute for Immunology and Inflammation, Faculty of Biology, Medicine and Health, University of Manchester, Manchester Academic Health Science Centre, Manchester M13 9PT, UK; richard.grencis@manchester.ac.uk; 3 MayBiotech SARL, 8 Rue de la Gendarmerie, 97620 Bouéni, France

**Keywords:** *Trichuris*, antiparasitic drugs, oxantel, nAChR, helminths, electrophysiology, *Xenopus* oocytes

## Abstract

The human whipworm, *Trichuris trichiura*, is estimated to infect 289.6 million people globally. Control of human trichuriasis is a particular challenge, as most anthelmintics have a limited single-dose efficacy, with the striking exception of the narrow-spectrum anthelmintic, oxantel. We recently identified a novel ACR-16-like subunit from the pig whipworm, *T. suis* which gave rise to a functional acetylcholine receptor (nAChR) preferentially activated by oxantel. However, there is no ion channel described in the mouse model parasite *T. muris* so far. Here, we have identified the ACR-16-like and ACR-19 subunits from *T. muris*, and performed the functional characterization of the receptors in *Xenopus laevis* oocytes using two-electrode voltage-clamp electrophysiology. We found that the ACR-16-like subunit from *T. muris* formed a homomeric receptor gated by acetylcholine whereas the ACR-19 failed to create a functional channel. The subsequent pharmacological analysis of the *Tmu*-ACR-16-like receptor revealed that acetylcholine and oxantel were equally potent. The *Tmu*-ACR-16-like was more responsive to the toxic agonist epibatidine, but insensitive to pyrantel, in contrast to the *Tsu*-ACR-16-like receptor. These findings confirm that the ACR-16-like nAChR from *Trichuris* spp. is a preferential drug target for oxantel, and highlights the pharmacological difference between *Trichuris* species.

## 1. Introduction

The human whipworm, *Trichuris trichiura*, is a Clade I parasitic nematode [1] and one of the Soil-Transmitted Helminths (STHs) which include the roundworm *Ascaris lumbricoides* and the hookworms *Ancylostoma duodenale* and *Necator americanus*. *Trichuris trichiura* is estimated to infect 289.6 million people globally, primarily living in low- and middle-income countries [2]. Infection with *T. trichiura* is rarely fatal, but affects the health and the nutritional status of the host [3,4]. High intensity infections are responsible for dysentery syndrome, anemia, finger clubbing and rectal prolapse [5]. Control of trichuriasis (and STHs in general) relies on hygiene education, access to appropriate sanitation and periodic mass drug administration of the anthelmintic drugs, albendazole and mebendazole which belong to the benzimidazoles anthelmintic drug class [6]. However, trichuriasis has proven notoriously difficult to control using a single-oral-dose treatment regime with these drugs [7,8,9,10,11,12] and there are only a few anthelmintic drugs effective against *Trichuris* infections. One of these is oxantel, which is a *m*-oxyphenol of pyrantel which belongs to the tetrahydropyrimidine chemical class. Oxantel was developed in 1972 [13] and marketed in 1974 as a veterinary, narrow-spectrum anthelmintic, specifically for the treatment of *Trichuris* [14]. Clinical trials have shown oxantel to be more efficient than albendazole and mebendazole for the control of trichuriasis [15,16,17,18].

*Trichuris muris* is a natural whipworm of rodents that bears some common features to *T. trichiura* and serves as a laboratory system to study human whipworm infection. *T. muris* is widely used as a model for anthelminthic screenings in vitro as well as for the clinical assessment of anthelmintic efficacy in vivo [19,20,21,22]. *T. muris* has several advantages as the adult worms are more accessible than *T. trichiura* obtained from humans, it has a shorter prepatent time than the pig whipworm *T. suis*, also used as a model for *T. trichiura* (i.e., 32 days versus 41–49 days) [23,24,25], hence obtaining *Trichuris* from mice is less labor- and cost intensive than from pigs. It is, therefore, important to identify and characterize anthelmintic drug targets from *T. muris* to further understand the mode of action of current compounds such as oxantel, and to evaluate novel anthelmintic compounds alone or in combinations.

Cholinergic anthelmintics, such as oxantel [26], exert their anthelmintic effect by paralyzing the worms which are subsequently expelled from the host or killed [27]. This effect is mediated by nicotinic acetylcholine receptors (nAChRs) which are pentameric proteins that are either homomeric- or heteromeric ligand-gated ion channels expressed in neuronal-, non-neuronal and muscle cell membranes [28,29]. Previously, no ion channel from Clade I parasitic nematodes had been characterized. Recently, we identified a preferential drug target for oxantel from the pig whipworm *T. suis*, and achieved its functional characterization in *Xenopus laevis* oocytes and the free-living nematode *Caenorhabditis elegans*. This drug target is a homomeric acetylcholine receptor (AChR), constituted of five ACR-16-like subunits that are specific to Clade I nematodes [30]. Due to the high oxantel sensitivity of this receptor, and in reference to the previously reported *L*-AChR, *N*-AChR, *B*-AChR and *M*-AChR (respectively for *L*evamisole-sensitive, *N*icotine-sensitive, *B*ephenium-sensitive and *M*orantel-sensitive) [31,32], we referred to this *Tsu*-ACR-16-like receptor as an *O*-AChR subtype.

*Caenorhabditis elegans* possesses one of the most extensive, and diverse nAChR gene family [33], with 29 nAChR subunits divided into five groups based on sequence homology and named after the first of their number to be discovered [34]. One of these groups, the ACR-16 group, contains 11 genes [34], which is in sharp contrast to the low number of nAChR subunits found within the ACR-16 group of Clade I parasitic nematodes. In our previous database search for a potential oxantel target within *Trichuris* spp., we identified only two nAChR subunits from the ACR-16 group of *Trichuris* spp. [30]. This was in accordance with Williamson et al. [35], who reported only two members from the ACR-16 group in the genomic data from the closely related Clade I parasitic nematode *Trichinella spiralis*. One was the ACR-16-like subunit, which constituted the *O*-AChR from *T. suis*, while the other was a clear orthologue of the ACR-19 subunit from *C. elegans* (*Cel*-ACR-19).

In contrast to *T. suis,* to date there are currently no ion channels of *T. muris* characterized and the respective roles of the ACR-16 group subunits have not yet been investigated in this important model species. Here, we aimed to characterize the ACR-16-like and ACR-19 subunits from *T. muris* at the functional level. We achieved the functional reconstitution of a *T. muris* oxantel-sensitive AChR in the *Xenopus* oocyte expression system and compared the pharmacological properties of the *O*-AChRs from *T. muris* versus *T. suis*. Our results show that the *O*-AChR subtype from *T. muris* is highly sensitive to oxantel, thus additionally explain the high efficacy and specificity of oxantel on whipworm species. Finally, our results highlight the pharmacological differences of the *O*-AChRs between *Trichuris* species and emphasize the lack of knowledge around parasitic receptor compositions.

## 2. Results

### 2.1. Identification of ACR-16-Like and ACR-19 Sequences from Trichuris muris

**Figure 1:**
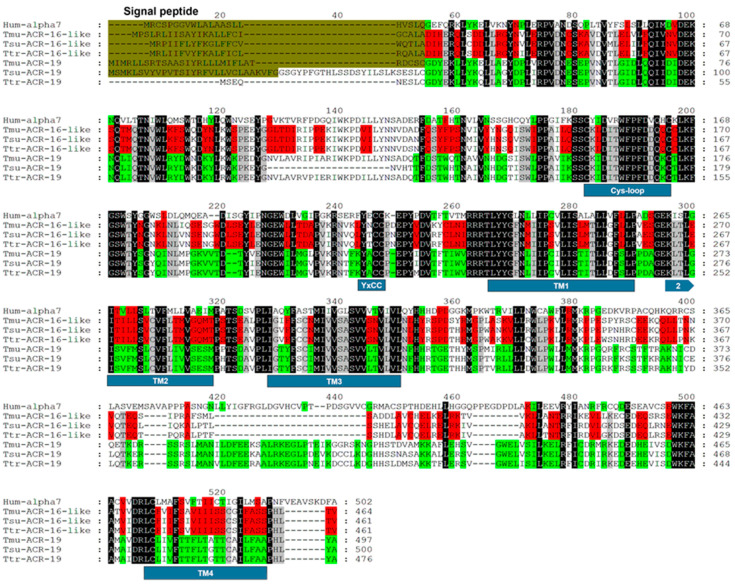
Amino-acid alignment of ACR-16-like and ACR-19 subunits from *T. muris, T. suis* and *T. trichiura* and the human α7 subunit Predicted signal peptide sequences are shaded in dark green, the Cys-loop, the transmembrane regions (TM1-TM4) and the YxCC motif is shown below the sequences. Conserved amino acids between ACR-16-like and ACR-19 subunit sequences are highlighted in black, conserved amino acids for ACR-16-like and ACR-19 are highlighted with red and green, respectively, nematode specific amino acids are highlighted in grey.

*Trichuris muris* is, as with *T. suis*, used as a model for *T. trichiura*. Oxantel was previously shown to act on the nAChR made of ACR-16-like subunits from *T. suis* [30]. To investigate the ACR-16-like nAChR from *T. muris*, we identified and cloned the full-length coding sequence of the *T. muris acr-16-like* subunit cDNA (*Tmu-acr-16-like*). The cloned *T. muris acr-16-like* sequence differed from the putative full-length *Tmu-acr-16-like* cDNA sequence available in WormBase (accession number WBGene00290200) by two synonymous nucleotide substitutions (CCG → CCA at codon 327 and AGT → AGC at codon 427), and was therefore deposited in GenBank under the accession number MZ322663. To explore all the members of the ACR-16 group of *Trichuris* spp, we performed searches in genomic databanks that led to the identification of the *C. elegans acr-19* gene orthologue in *T. muris* [30]. Consequently, the full-length *Tmu-acr-19* cDNA sequence was amplified by PCR and subsequently cloned and sequenced. Our clone was identical to the putative full-length *Tmu-acr-19* cDNA sequence available in WormBase (accession number WBGene00291941). An amino-acid alignment of the *Tmu*-ACR-16-like and *Tmu*-ACR-19 sequences with their related counterparts from *T. suis* and *T. trichiura* is shown in Figure 1. The nematode ACR-16 nAChR subunit has been described as a homolog of the mammalian α7 nAChR subunit, for comparison, the amino-acid sequence of the human α7 subunit is therefore included in the alignment [36,37,38]. The *Tmu*-ACR-16-like subunit is 464 amino acids long and shares 90 and 91% identities with the ACR-16-like subunits from *T. suis* and *T. trichiura*, respectively. For comparison, the ACR-16-like subunits from *T. suis* and *T. trichiura* share 93% identities. The *Tmu*-ACR-16-like subunit shares 42% identities with the human α7 subunit. The *Tmu*-ACR-19 subunit is 497 amino acids long, and shares 78 and 88% identities with the ACR-19 subunits from *T. suis* and *T. trichiura*, respectively. The ACR-16-like- and the ACR-19 subunit sequences from *T. muris* and *T. suis* all share typical features of nAChR subunits including a predicted signal peptide, four transmembrane areas (TM1-TM4), a Cys-loop motif and the YxCC motif that characterizes an α-type nAChR receptor subunit. The extracellular domain (ECDs) ligand binding loops A-E of the ACR-16-like subunits from *Trichuris* spp. and the ACR-16 subunits from Clade III (*Ascaris suum, Parascaris equorum*) and Clade V (*C. elegans*, *A. ceylanicum, A. caninum, N. americanus*, *Haemonchus contortus*) nematodes [1] are shown in Figure S1. The ECDs are based on a homology model of the ECDs of the ACR-16 subunit from *A. ceylanicum*, *N. americanus*, *C. elegans*, and *T. muris* performed by Kaji et al. [38]. The ligand binding loops of the ACR-16-like subunit from *Trichuris* spp. are in general different from Clade III and V nematodes. However, among the *Trichuris* spp. the ligand binding loops of the *Tmu*-ACR-16-like subunit is the most divergent. In total, the ACR-16-like subunit of *T. muris* has seven residues in loop E, F, and C, respectively, which differ from the corresponding residues in the ACR-16-like subunit of *T. suis* and *T. trichiura* (i.e., loop E: Tyr138 and Gly140 instead of His and Ser, loop F: Ser189 instead of Asn, loop C: Lys210, Lys214, Asn217, and Asp221 instead of Ile, Gln, Thr, and Asn, respectively, Figure S1). In contrast, the binding loops of *T. suis* and *T. trichiura* are highly similar, with only one residue in loop E that differs (Met144 in *T. suis* instead of Val in *T. muris* and *T. trichiura*).

### 2.2. The T. muris ACR-16-Like Subunit Forms a Functional Acetylcholine Receptor in Xenopus laevis Oocytes

**Figure 2:**
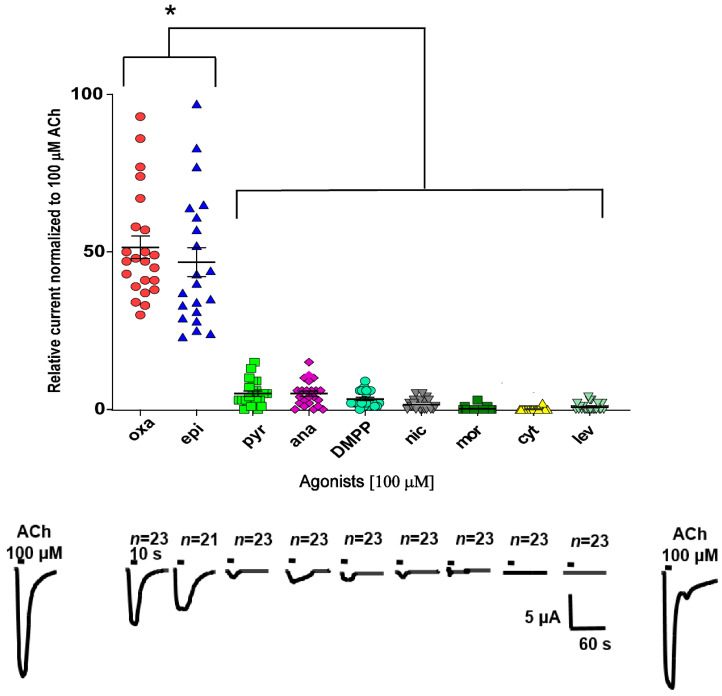
Effect of anthelmintics and cholinergic agonists on the *Tmu*-ACR-16-like receptor. A scatter dot plot (mean ± SEM) and representative sample traces show the rank order efficacy in μA of four cholinergic anthelmintics: oxantel (oxa), pyrantel (pyr), morantel (mor), levamisole (lev) and five nicotinic agonists: epibatidine (epi), anabasine (ana), dimethyl phenyl piperazine (DMPP), nicotine (nic) and cytosine (cyt). * *p* < 0.02. The number of oocytes (*n*) used for each agonist is given above the traces, the perfusion time of each compound was 10 s as indicated with short bars above the traces.

**Figure 3:**
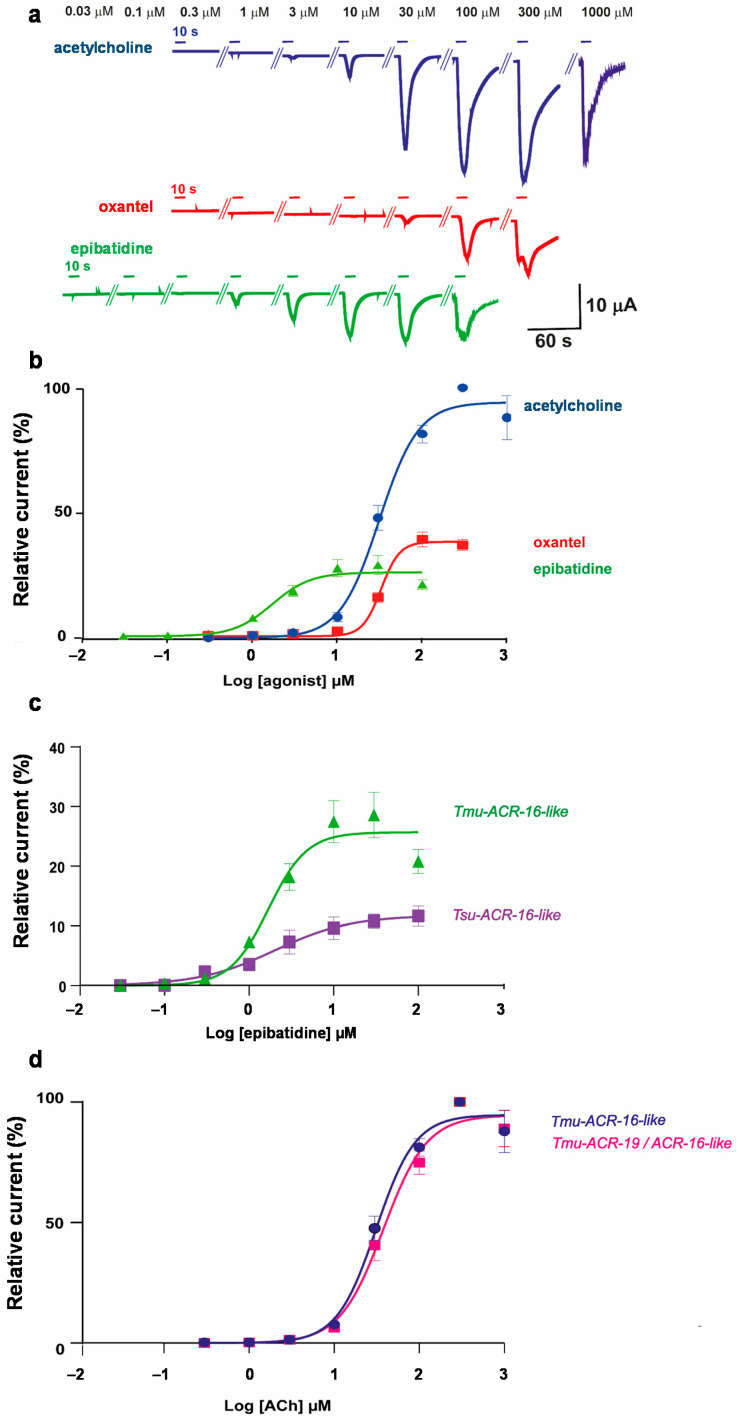
Concentration-response relationships. (**a**) Representative current traces of individual *Xenopus* oocytes expressing the *Tmu*-ACR-16-like receptor challenged with increasing concentrations of ACh (blue), oxantel (red) and epibatidine (green). Compounds were applied for 10 s (short bars); (**b**) Concentration-response curves of ACh, oxantel and epibatidine on the *Tmu*-ACR-16-like receptor; (**c**) Concentration-response curves for epibatidine on the ACR-16-like receptor from *T. muris* (also shown in **a**) and *T. suis*; (**d**) Concentration-response curves for ACh on eggs microinjected either with *Tmu-acr-16-like* cRNA (also shown in **a**) or with *Tmu-acr-16-like* and *Tmu-acr-19* cRNAs. All current responses are normalized to current responses induced by 300 μM ACh and given as mean ± SEM.

To investigate potential functional nAChRs made of *T. muris* ACR-16-like and ACR-19 subunits, we microinjected their corresponding cRNAs in *X. laevis* oocytes and responses to agonists were subsequently monitored using two-electrode voltage-clamp (TEVC) electrophysiology. The cRNA of the *X. laevis* chaperone protein *R*esistance to *I*nhibitors of *C*holinesterase *ric-3* was also added to the respective subunit cRNAs as it is required to promote the expression of nAChRs [30,37,38,39,40,41]. We found that injections of the *Tmu-acr-16-like* cRNA led to the robust expression of a functional homomeric receptor, which elicited a response in the μA range when exposed to 100 μM acetylcholine (ACh) (Figure 2 and Figure 3a). In contrast to injection with the *Tmu-acr-16-like* cRNA, the injections of *Tmu-acr-19* cRNAs never led to recordings of ACh-elicited currents (*n* = 14), indicating that this subunit did not form a functional homomeric ACh-sensitive channel. To pharmacologically characterize the *Tmu*-ACR-16-like receptor, we assayed four cholinergic anthelmintics (i.e., oxantel, pyrantel, morantel and levamisole) and five nicotinic agonists (i.e., epibatidine, anabasine, nicotine, dimethyl phenyl piperazine (DMPP) and cytisine) all at a concentration of 100 μM. For each oocyte, all responses induced by each compound, were normalized to the response of 100 μM ACh. The relative current responses of these compounds are given in Figure 2 as a scatter dot plot with normalized means ± SEM, along with representative traces and the number of oocytes (*n*) used for each agonist. When oxantel and epibatidine were applied onto the oocytes, we could record large µA currents corresponding to 51.5 ± 3.6% and 48.3 ± 5.0% of the 100 µM ACh response, respectively. Interestingly, oxantel and epibatidine induced significantly higher current responses on the *Tmu*-ACR-16-like receptor than any of the other agonists tested (*p* < 0.02), including pyrantel (4.6 ± 0.8%), which chemical structure is very similar to oxantel. Levamisole, cytisine, and morantel did not elicit any current. The rank order potency series for the compounds tested was oxantel = epibatidine > pyrantel ~ anabasine ~ DMPP ~ nicotine > morantel = cytosine = levamisole thus showing that this receptor is different from nicotine, pyrantel, and levamisole receptor subtypes described in other nematode species.

### 2.3. Characterization of ACh, Oxantel, and Epibatidine Effects on T. muris and T. suis ACR-16-Like AChRs

Oxantel has previously been shown to act as a full agonist on the ACR-16-like receptor from *T. suis*, whereas epibatidine only induced a minor response. Full concentration-response experiments were carried out to explore the ACR-16-like channels sensitivities to ACh, oxantel, and epibatidine from *T. muris* versus *T. suis*. Concentration-response relationships for ACh, oxantel and epibatidine on the *Tmu*-ACR-16-like receptor are shown in Figure 3a,b. The ACh concentration-response curve was characterized by an EC_50_ value of 31.1 ± 0.3 μM (mean ± SEM, *n* = 16). The highest current response was measured with 300 μM ACh, hence, all other measurements were normalized to the current response at 300 μM ACh. We found that the oxantel EC_50_ value of the *Tmu*-ACR-16-like receptor was 32.7 ± 0.3 μM (*n* = 18) suggesting that oxantel and ACh were equally potent. Surprisingly, epibatidine was found to be the most potent compound on the *Tmu*-ACR-16-like receptor with an EC_50_ of 1.7 ± 0.4 μM (*n* = 11). Acetylcholine had the highest efficacy with an *I_max_* (mean ± SEM) of 94.7 ± 0.7% followed by 37.9 ± 0.3% for oxantel and 25.7 ± 0.5% for epibatidine (*p* < 0.0001). To compare the pharmacological properties of the ACR-16-like receptor from *T. muris* to that of *T. suis*, we further performed the concentration-response relationship for epibatidine on the *Tsu*-ACR-16-like receptor. Figure 3c shows the comparison of the concentration-response relationship for epibatidine on the ACR-16-like receptor from *T. muris* and *T. suis*. The efficacy, *I_max_*, of epibatidine was higher, 25.7 ± 0.5% (*n* = 11) for the *Tmu*-ACR-16-like receptor as compared to 11.1 ± 0.5% (*n* = 8) for the *Tsu*-ACR-16-like receptor (*p* = 0.02). In addition, the concentration-response curve revealed an epibatidine EC_50_ value of 1.9 ± 0.5 μM (*n* = 8) for the *Tsu*-ACR-16-like receptor that was in the same range as the one from the *Tmu*-ACR-16-like receptor (1.7 ± 0.4 μM, *n* = 11).

The ACR-16-like and the ACR-19 both belong to the ACR-16 group of nAChR subunits; however, only the *Tmu*-ACR-16-like subunits gave rise to a functional AChR. Because co-expressions of closely related AChR subunits, such as DES-2 and DEG-3 from *C. elegans* [35], as well as ACR-26 and ACR-27 from the livestock strongyle *H. contortus* and the horse ascarid parasite *P. equorum* [27], have been described as a requirement for the reconstitution of functional heteromeric AChRs, we hypothesized that the ACR-16-like and ACR-19 subunits from *T. muris* could assemble into a heteromeric receptor. Therefore, we microinjected equimolar concentrations of *Tmu-acr-16-like* and *Tmu-acr-19* cRNAs (together with *Xla-ric-3*) in *X. laevis* oocytes. Co-expression of *Tmu-acr-16-like* and *acr-19* produced functional channels that responded to ACh and displayed an ACh EC_50_ value of 37.9 ± 0.5 μM (*n* = 6) which was not significantly different from the EC_50_ value obtained for the homomeric *Tmu*-ACR-16-like receptor (31.1 ± 0.3 μM, *n* = 16). The concentration-response relationships of ACh (0.3 μM–1 mM) on the putative *Tmu*-ACR-19/*Tmu*-ACR-16-like- and the *Tmu*-ACR-16-like receptor are shown in Figure 3d. These results could suggest that *Tmu*-ACR-16-like does not assemble into functional heteromultimers with *Tmu*-ACR-19.

## 3. Discussion

In the present study, we report for the first time the identification and the expression of a functional AChR from the model parasitic nematode *T. muris*. Indeed, there was to our knowledge no functional characterization of any ion channels from *T. muris* reported to date. We showed that the amino-acid sequence of the *Tmu*-ACR-16-like subunit is highly conserved with the *T. suis* orthologue. As with the *T. suis O*-AChR, we found that the *Tmu*-ACR-16-like subunit gave rise to a functional homomeric AChR highly sensitive to oxantel when expressed in *Xenopus* oocytes. These results additionally explain the high efficacy and specificity of oxantel on whipworm species, and suggest that *T. muris* is a valid pharmacological model for *T. trichiura* at least in relation to oxantel.

Most importantly, we also report that the pharmacology of the ACR-16-like receptor from the genus *Trichuris* is not identical, highlighting a putative different pharmacological profile of *T. muris* and *T. suis*. These differences emphasize the importance of species-specific drug-target characterization and caution in transferring data interpretation between *Trichuris* spp.; perhaps these differences even suggest that drug discovery programs should be species-specific.

Here, we found that oxantel acted as a partial agonist with similar potency as ACh, epibatidine acted as a partial agonist with a higher potency than ACh, whereas pyrantel induced only a minor response on the *Tmu*-ACR-16-like receptor. These findings emphasize species-specific differences at the receptor level, since oxantel acted as a full-, pyrantel as a partial-, and epibatidine as a weak agonist on the *Tsu*-ACR-16-like receptor [30]. Oxantel was predicted to bind to the ACR-16 of *T. muris* (*Tmu*-ACR-16-like) with high affinity in a recent in silico ligand binding analysis of the extracellular domains of nematode ACR-16 nAChRs [38]. Our results support these predictions, but also show that oxantel acts as a partial agonist on the *Tmu*-ACR-16-like receptor. Oxantel may therefore exert its anthelmintic effect by reducing either the potency or the efficacy of the natural ligand ACh. Nevertheless, oxantel is more efficient than the cholinergic anthelmintics, pyrantel, levamisole, and monepantel against *T. muris* in vitro [19,20,22] and has excellent trichuricidal properties in mice [20]. As the potency of oxantel was similar to ACh in our study, and presuming that the *Tmu*-ACR-16-like receptor is the major oxantel target in *T. muris*, we therefore refer to the *Tmu*-ACR-16-like receptor as an *O*-AChR subtype similar to the *O*-AChR from *T. suis*. To our knowledge, there are only a few studies investigating the effect of oxantel on mammalian nAChRs. A recent in vitro receptor binding assay reports oxantel to bind to the human α7 with an estimated maximal inhibitory concentration (IC_50_) of 3.48 μM. A similar binding assay was performed using the whole brain membranes (minus cerebellum) from Wistar rats, which contains nAChR, here an estimated IC_50_ value of 33 μM was reported [42]. For comparison, the IC_50_ value of oxantel pamoate for the larvae stage 4 (L4) of *T. muris* has previously been reported to be 3.9 μM [20], which by the authors is categorized as in the same order as the IC_50_ values for the mammalian nAChRs. It should be noted that the authors emphasize the IC_50_ values of the nAChRs to be interpreted with caution as data are insufficient [42]. The α7 subunit from chicken has been expressed in *X. laevis* oocytes and assayed with oxantel [43]. An approximate EC_50_ value of 80 μM oxantel was found, which is considerably higher than for the *Tmu*-ACR-16-like receptor (i.e., 32.7 ± 0.3 μM).

The pamoate salt of oxantel, used in treatment of human trichuriasis, limits the absorption of oxantel, which is stated to be approximately 8–10%, allowing the anthelmintic to reach therapeutic levels in the lower part of the intestine where *Trichuris* spp. are situated [14,44]. Permeability of intestinal epithelial to oxantel pamoate has been evaluated in vitro using Caco-2 cells. The mean apical to basolateral and basolateral to apical permeability was 0.2 and 0.4 × 10^−6^ cm/s, respectively. This was in the same range as colchicine and was therefore by the authors considered to be low permeability [42]. The poor gastrointestinal drug absorption has been evaluated in vivo by Cowan et al. [45] who found the plasma level concentrations to be below the limit of quantification after oral administration of 100 mg/kg oxantel pamoate to mice, the poor systemic bioavailability has also been confirmed in rats [46]. Non-detectable levels of oxantel pamoate in the plasma may be due to metabolism of the parent compound. However, oxantel pamoate has been found to be metabolic stable when incubating oxantel pamoate with human and rat intestinal microsome. Due to the metabolic stability and poor absorption, hepatic metabolism has not been further evaluated [42]. It is assumed that oxantel pamoate primarily stays in the intestinal tract; however, further pharmacokinetic studies are suggested for Phase I studies [42]. The clinical safety of oxantel pamoate has been evaluated in a parallel, randomized, placebo-controlled, single-blind trial using six different dose levels between 5–30 mg/kg. In this study, the frequency of side effects seemed to be independent of the oxantel pamoate dose [18]. According to Quantrel^®^ (oxantel pamoate-pyrantel pamoate) package information leaflet, side effects, if occurring, are usually related to the gastrointestinal tract. This is in line with oxantel pamoate primarily stays in the gastrointestinal tract where it is believed to interact with nAChRs located in human intestinal cells. However, Palmeirim et al. (2021) [42] reviewed clinical trials implemented after 2000, and reported that the most common side effects described were stomach pain and headache.

Epibatidine is a toxic alkaloid, isolated from the skin of the Ecuadorian poison frog *Epipedobates tricolor* used by indigenous tribes in darts for hunting [47]. The great interest in epibatidine arose with the discovery of its analgesic effect mediated by non-opioid receptors [48]. Epibatidine interacts with nAChRs in the neuro-muscular junction and the central nervous system [49], but due to its high toxicity in mammals it is not desirable as a future anthelmintic compound. However, it is interesting to note that the sensitivity of the ACR-16-like receptor to epibatidine varies between *Trichuris* spp. emphasizing the importance of species-specific receptor characterization. Pyrantel-induced activation has been associated with glutamic acid in ligand binding loop B [50,51] and a glutamine in loop D [50]. Recently it was noted by Kaji et al. [38] that all characterized ACR-16 subunits lack these residues and instead possess an analogous loop D aspartic acid and loop B glycine. These residues could therefore explain the lack of efficacy to pyrantel but not the differential sensitivities to pyrantel between ACR-16 nAChRs from the zoonotic hookworm of dogs, *A. ceylanicum*, and the human hookworm *N. americanus*. The divergent ACR-16-like subunits of *T. muris* and *T. suis* possess only the loop B glycine (position 179 in Figure S1), but have an analogous loop D lysine (position 82 in Figure S1). The lysine could explain why the ACR-16-like receptor of *Trichuris* spp. has a different sensitivity to pyrantel, but in line with Kaji et al., this residue cannot explain why the ACR-16-like receptor from *T. muris* is less sensitive to pyrantel (mean ± SEM, 4.6 ± 0.8%) than the ACR-16-like receptor from *T. suis* (26.1 ± 2.1%) [30]. Our results therefore suggest that additional residues may be involved in pyrantel-induced activation. However, it is important to recall that pyrantel has no effect against adult *Trichuris* [13], and that pyrantel preferentially targets another AChR subtype made of the subunits UNC-29, UNC-38 and UNC-63 isolated from the pig nodular worm *Oesophagostomum dentatum* [52].

The pharmacological differences between the ACR-16-like receptors of *T. muris* and *T. suis* may be attributed to several different residues within the ligand binding loops. In general, the ligand binding loops of the *Tmu*-ACR-16-like subunit diverge the most from *T. suis* and *T. trichiura*, whereas the binding loops of *T. suis* and *T. trichiura* are highly similar, with only one residue in loop E that differs (Met144 in *T. suis* instead of Val in *T. muris* and *T. trichiura*). This valine has previously been speculated to accommodate more space to oxantel within the binding pocket [38], but as *T. suis* has Met163 and oxantel acts as a full agonist on the *Tsu*-ACR-16-like receptor, it is more likely that other residues in the binding loops are of greater importance in relation to oxantel binding. However, the high identities of the ACR-16-like subunits (i.e., 93%) from *T. suis* and *T. trichiura*, and particularly the high similarities of the ligand binding loops suggest a comparable pharmacology of these species, at least in relation to the ACR-16-like receptor. Future pharmacological studies on the ACR-16-like receptor from *T. trichiura* would provide important information on the amino acids involved in the sensitivity to oxantel.

The attempt to reconstitute a functional AChR using the nematode ACR-19 subunit has so far never been described, even in the model nematode *C. elegans*. Here, we explored for the first time whether the *Tmu*-ACR-19 subunit could form either a functional homomeric- or a heteromeric receptor when expressed in *X. laevis* oocytes either alone, or in combination with *Tmu*-ACR-16-like subunits. We observed no difference in ACh sensitivities between the eggs injected with *Tmu-acr-16-like* alone and the eggs co-injected with *Tmu-acr-16-like/acr-19* cRNAs. We therefore proposed that the current responses were due to the functional homomeric *Tmu*-ACR-16-like receptor and not a heteromeric ACR-19/ACR-16-like receptor. However, we cannot rule out the possibility that there might be a heteromeric ACR-19/ACR-16-like receptor with a sensitivity to ACh similar to the one of the ACR-16-like receptor. More pharmacological and biochemical investigations will be needed to address this hypothesis. The reason for lack of receptor functionality is presently unclear. There is currently a lack of knowledge of which nAChR subunit(s) and/or ancillary and/or auxiliary proteins are required to form a functional receptor including the ACR-19 subunit. Upstream regulation of *acr-19* expression by *acr-15* has been reported for *C. elegans* in response to chronic nicotine exposure [53]. It is, therefore, possible that some functional association exists between *acr-15* and *acr-19* in *C. elegans*. The ACR-15 subunit is in the ACR-16 group of *C. elegans* [54]; however, we and others have found an *acr-15* subunit in the genome of Clade I parasitic nematodes [35]. It is, therefore, unlikely that ACR-15 is required for the expression of a functional receptor including the ACR-19 subunit in *Trichuris* spp. The nAChRs from numerous organisms, including ***C. elegans***, have been shown to require various ancillary or auxiliary proteins such as RIC-3, UNC-50 (***UNC***oordinated-50) and UNC-74 (***UNC***oordinated-74), EAT-18 and MOLO-1 (***MO***dulator of ***L***evamis***O***le receptor-1) for functional expression [39,55,56,57]. In this study, we only used the ancillary protein RIC-3, hence, we cannot exclude the requirement of some of the above-mentioned, or yet unidentified, ancillary or auxiliary proteins.

## 4. Materials and Methods

### 4.1. Ethic Statement

Animal experiments were performed under the regulations of the UK Home Office Scientific Procedures Act (1986) and were subject to local ethical review by the University of Manchester Animal Welfare and Ethical review Body (AWERB) and followed ARRIVE guidelines.

### 4.2. Experimental Animals and Infections

The worm material used in this study was of the Edinburgh strain, originally obtained from The Wellcome Research Laboratories, London [58], and was isolated from 6–8-week-old male Severe Combined Immunodeficient (SCID) mice infected with 400 *T. muris* eggs by oral gavage. Forty-two days later, mice were killed using CO_2_ and the cecum and proximal colon were removed. The cecum and colon were split and washed in RPMI 1640 medium plus 500 U/mL penicillin and 500 µg/mL streptomycin (Sigma-Aldrich UK). Adult worms of both sexes were removed using fine forceps and washed using the above medium three times before being placed in 100% ethanol.

### 4.3. Drugs

All drugs were purchased at Sigma-Aldrich (France). Stock solutions were made in either recording solution (100 mM NaCl, 2.5 mM KCl, 1 mM CaCl_2_·2H_2_O, 5 mM HEPES, pH 7.3) or DMSO (100%) and stored at either −20 or 4 °C until use. Before use, stock solutions were dissolved in recording buffer with a maximum final concentration of DMSO of 0.1%.

### 4.4. Sequence Analysis and Alignment

The *Tsu*-ACR-16-like (accession number **MT386096**) and the *Tsu*-ACR-19 (accession number KFD70086) were used as queries in database searches for amino-acid sequences of ACR-16-like and ACR-19 from *T. muris* using the protein-protein BLAST (BLASTp) service at the National Center for Biotechnology Information (NCBI) service [59]. The *Tsu*-ACR-16-like and the *Tsu*-ACR-19 were used in an alignment with the identified sequences. The accession numbers of the identified sequences used for the alignment are: *Trichuris muris*: ACR-16-like **MZ322663**; ACR-19 WBGene00291941. *Trichuris suis*: ACR-16-like **MT386096**; ACR-19 KFD70086. *Trichuris trichiura*: ACR-16-like CDW52185; ACR-19 CDW53523. *Homo sapiens*: α7 subunit isoform 1 P36544. Signal peptide was predicted using the SignalP 4.1 server [60] and the transmembrane regions were predicted using the TMHMM version 2 server [61]. Deduced amino-acid sequences were aligned using the MUSCLE algorithm [62] and additionally processed in GeneDoc (IUBio). The accession numbers of the ACR-16-like and ACR-16 sequences from Clade I, III and V nematodes used for the alignment to show the ligand binding loops in the ECDs are: *Trichuris muris*: **MZ322663**; *Trichuris suis*: **MT386096**; *Trichuris trichiura*: CDW52185; *Ascaris suum*: **KP756901**; *Parascaris equorum*: AZS27834.1; *Caenorhabditis elegans*: CCD64102.1; *Haemonchus contortus*: AZS27833.1; *Ancylostoma ceylanicum*: MT163735; *Ancylostoma caninum*: QEM53385; *Necator americanus*: MT163736.

### 4.5. Cloning of the Coding Sequence of the ACR-16-Like and ACR-19 Subunits from Trichuris muris

Total RNA was extracted from *T. muris* using NucleoSpin RNA XS kit and subsequently treated with rDNase (Macherey-Nagel, France). First strand cDNA synthesis was performed with 0.8 μg of total RNA using the Maxima H minus Reverse Transcriptase kit (Thermo Scientific, MA, USA) according to the manufacturers’ recommendations. The full-length coding sequences of *Tmu-acr-16-like* and *Tmu-acr-19* were obtained with nested PCRs using the Phusion High Fidelity Polymerase (New England BioLabs, MA, USA) and the first strand cDNA as a template. Primer sequences were designed based on predicted coding sequences of the *Tmu-acr-16*-*like* subunit (WBGene00290200), and the putative *Tmu-acr-19* (WBGene00291941) available in Wormbase Parasite, version VBPS15 (WS276). The first round of PCRs was performed with the primer-combination F0/R0 and the second round with F1/R1 of which the latter contained the *XhoI* and *ApaI* restriction enzyme sites and a 11–14 bp nucleotide overhang complimentary to 11–14 bp in the linearized expression vector pTB-207. The primer pair sequences were as followed for *Tmu-acr-16*-*like* and the putative *Tmu-acr-19*, respectively: F0-*Tmu-acr-16*-*like*-5′-agataggacagttgcgaggag-3′, R0-*Tmu-acr-16*-*like*-5-agaatgccaaagagctcacc-3′, F1*-Tmu-acr-16*-*like*-pTB207-*XhoI*-5′-gcggccgctcgagatgccatcattgcggctgat-3′, R1-*Tmu-acr-16*-*like*-pTB207-*ApaI*-5′-gttctccccatttaaccgtgtaagggcccgagcttgatctggt-3′ and F0-*Tmu-acr*-19-5′-accattagcggcagaccgc-3′, R0-*Tmu-acr*-19-5′-tcaggaccaatttatgacgag-3′, F1-*Tmu-acr*-19-5′-ctggcggccgctcgagatgatcatgagattactcagcc-3′, R1-*Tmu-acr*-19-5′-accagatcaagctcgggcccctaggcatataaatgcggagc-3′. The purified PCR products were cloned into the expression vector pTB-207 using the In-Fusion HD Cloning kit (Clontech). Constructs were verified by sequencing, and the pTB-207-*Tmu-acr-16-like* linearized with the restriction enzymes *PaeI* and *NheI* and the pTB-207-*Tmu-acr-19* with *NehI*. cRNAs synthesis were performed using mMessage mMachine T7 transcription kit (Ambion), purified on NucleoSpin RNA columns (Machery–Nagel) and kept at −80 °C until use.

### 4.6. Electrophysiological Experiments in Xenopus laevis Oocytes

*Xenopus laevis* oocytes were purchased from Ecocyte Biosciences (Castrop-Rauxel Germany) and kept at 19 °C in incubation solution (100 mM NaCl, 2 mM KCl, 1.8 mM CaCl_2_·2H_2_O, 1 mM MgCl_2_·6H_2_O, 5 mM HEPES, 2.5 mM C_3_H_3_NaO_3_, supplemented with 100 U/mL penicillin and 100 μg/mL streptomycin, pH 7.3). The requirement of the chaperone protein RIC-3 for functional expression of the ACR-16-like receptor of *T. suis* has previous been described ([30]), hence, we co-injected each oocyte with 5 ng cRNA of *Xla-ric-3* and 25 ng cRNA of either *Tmu-acr-16-like*, *Tmu-acr-19*, *Tsu-acr-16-like*, or *Tmu-acr-16-like* and *Tmu-acr-19* in a total volume of 37 nL using. The oocytes were incubated for 3–5 days in incubation solution to allow for receptor expression. The function and the pharmacological properties of the resultant receptors was explored 3–5 days after microinjection on oocytes clamped at −80 mV using two-electrode voltage-clamp recordings obtained with the fully automated system Robocyte2 (Multichannel systems MCS GmbH, Germany). All experiments were performed with oocytes pre-incubated in 100 μM BAPTA-AM for 3.5 h in order to chelate intracellular Ca^2+^ ions and hereby prevent activation of endogenous calcium activated chloride channels during recordings [63]. The drug efficacy test was performed using 3 individual oocyte batches (*n* = 7–10 per batch). Each oocyte was initially exposed to 100 μM ACh for 10 s and subsequently to 100 μM of each of the 4 cholinergic anthelmintics; oxantel, pyrantel, morantel and levamisole, and the 5 nicotinic agonists; epibatidine, anabasine, DMPP, nicotine, and cytosine for 10 s each. Between each drug application, a wash-out period of 2 min with recording solution was applied. For individual eggs, peak current responses of each agonist was normalized to the peak current response induced by 100 μM ACh. The dose-response tests with the *Tmu*-ACR-16-like receptor were performed using 2 individual oocyte batches (*n* = 7–11 per batch), whereas the dose-response tests with the ACR-16-like receptor from *T. suis*, and the putative heteromeric *Tmu*-ACR-16-like/*Tmu*-ACR-19 receptor were performed on a single batch of oocyte (*n* = 6–8). First, the dose-response relationship of ACh was established at concentrations between 0.3 μM–1 mM. The current response, at which the maximum mean peak current to ACh was obtained, was used to normalize all other responses of ACh (0.3 μM–1 mM), oxantel (0.3–300 μM) and epibatidine (0.03–30 μM). Consequently, each oocyte exposed to oxantel and epibatidine was first challenged with 300 μM ACh.

### 4.7. Statistical Analysis

All acquired electrophysiological data were analyzed with Clampfit 10.7 (Molecular Devices, Sunnyvale, CA, USA) and GraphPad Prism 9 (GraphPad Software, La Jolla, CA, USA). All data analysis was performed on normalized current values. For the efficacy tests, the normalized drug-group means were statistically analyzed using a non-parametric Kruskal–Wallis Test with a Dunn’s multiple comparison test, where *p* < 0.05 was considered significant. The dose-response relationships were established by fitting the normalized currents as a function of drug concentration to a Hill equation using non-linear regression analysis with a variable slope model. The following equation was used:

*I*_rel_ = *I*_min_ + (*I*_max_ − *I*_min_)/(1 + 10^((LogEC_50_ − [*D*])*n_H_))

where *I*_rel_ is the mean relative current, *I*_max_, is the relative current obtained at saturating agonist concentration, *I*_min_ is the relative current obtained at agonist concentrations 0 µM, EC_50_ is the concentration of agonist at which 50% of the maximal current response is obtained, [*D*] is the drug concentration and *n*_H_ is the Hill coefficient. *I*_max_, EC_50_, and *n*_H_ were fitted as free parameters whereas *I*_min_, was constrained to 0.

## 5. Conclusions

In summary, this study confirms that the ACR-16-like receptor from *T. muris* is a preferential drug target for oxantel, thus an *O*-AChR subtype such as the ACR-16-like receptor from *T. suis*. Our data therefore support previous findings, which provided new insight for the high efficacy and specificity of oxantel on whipworms and emphasize the potential importance of species-specific pharmacological characterization for future anthelmintic discovery programs.

## Data Availability

Data sharing not applicable.

## Supplementary Materials

The following are available online at https://www.mdpi.com/article/10.3390/ph14070698/s1, Figure S1: Amino-acid alignment of the ACR-16-like subunit from *T. muris* (*Tmu*), *T. suis* (*Tsu*) and *T. trichiura* (*Ttr*) and the ACR-16 subunits from the Clade III parasitic nematodes *A. suum* (*Asu*), *P. equorum* (*Peq*) and the Clade V nematodes *C. elegans* (*Cel*), *A. ceylanicum* (*Ace*), *A. caninum* (*Aca*), *N. americanus* (*Nam*) and *H. contortus* (*Hco*). Conserved amino acids for the ACR-16-like from *Trichuris* spp. are highlighted with red. The extracellular domain (ECDs) ligand binding loops A-E are highlighted in green. * (in black) indicate amino acids residues within the binding loops of the ACR-16-like subunit from *T. muris* which differ from *T. suis* and *T. trichiura*. * (in red) indicate the amino acids residue within the binding loop of the ACR-16-like subunit from *T. suis* which differ from *T. muris* and *T. trichiura*. Predicted signal peptide sequences are shaded in dark green, the transmembrane regions (TM1-TM4) are shown below the sequences.
