# Supplementary figures and images for: Functional Characterization of the Oxantel-Sensitive Acetylcholine Receptor from Trichuris muris
Source: Pharmaceuticals (Basel). 2021 Jul 20;14(7):698. doi: 10.3390/ph14070698 (PMC8308736; doi:10.3390/ph14070698)

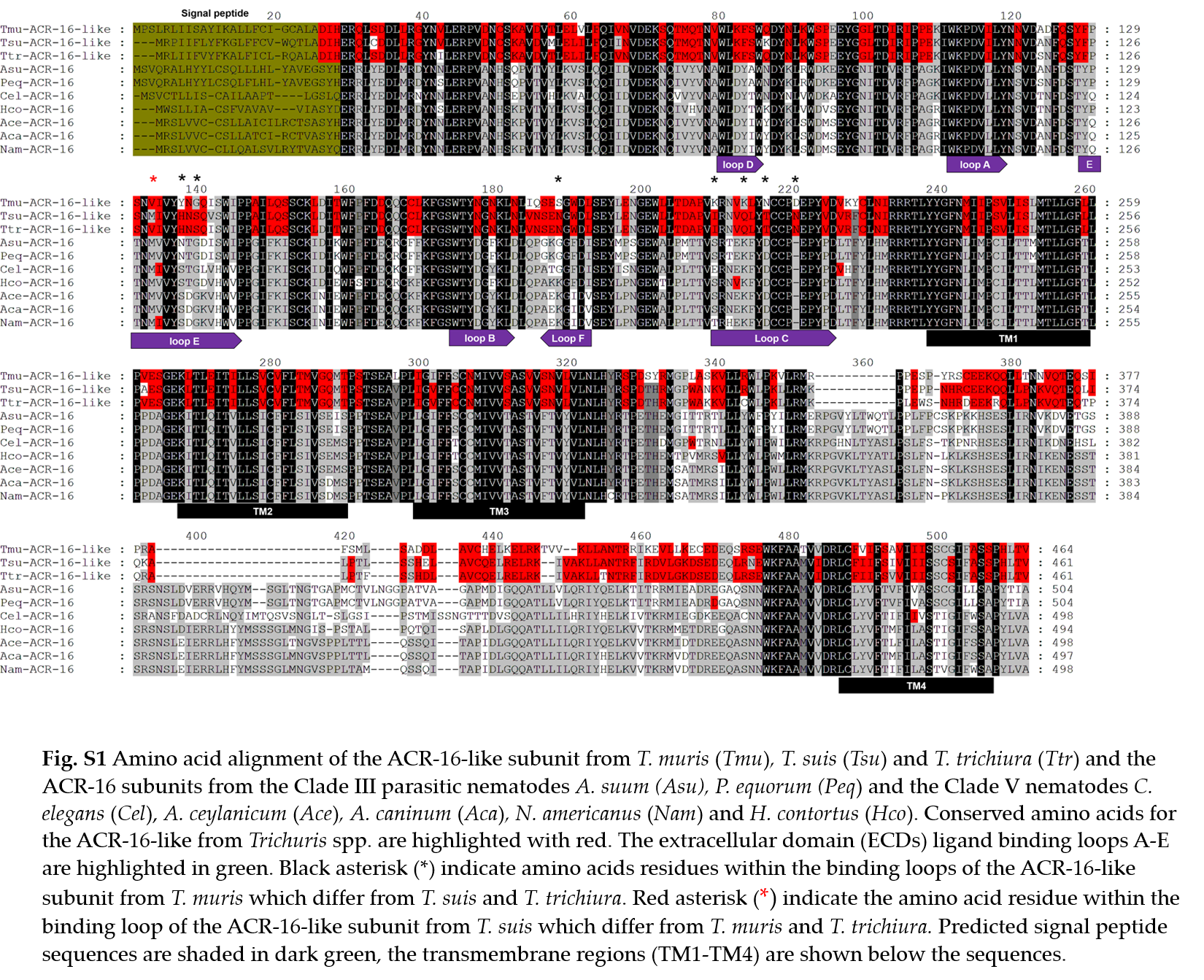

Supplement: Supplementary file 1 [file pharmaceuticals-14-00698-s001.zip › FigS1.png]
